# Utility of the Ba/F3 cell system for exploring on‐target mechanisms of resistance to targeted therapies for lung cancer

**DOI:** 10.1111/cas.15263

**Published:** 2022-01-23

**Authors:** Takamasa Koga, Kenichi Suda, Tetsuya Mitsudomi

**Affiliations:** ^1^ Division of Thoracic Surgery Department of Surgery Faculty of Medicine Kindai University Osaka‐Sayama Japan

**Keywords:** acquired resistance, adenocarcinoma of lung, Ba/F3, secondary mutation, tyrosine kinase inhibitor

## Abstract

Molecular targeted therapies are the standard of care for front‐line treatment of metastatic non‐small‐cell lung cancers (NSCLCs) harboring driver gene mutations. However, despite the initial dramatic responses, the emergence of acquired resistance is inevitable. Acquisition of secondary mutations in the target gene (on‐target resistance) is one of the major mechanisms of resistance. The mouse pro‐B cell line Ba/F3 is dependent on interleukin‐3 for survival and proliferation. Upon transduction of a driver gene, Ba/F3 cells become independent of interleukin‐3 but dependent on the transduced driver gene. Therefore, the Ba/F3 cell line has been a popular system to generate models with oncogene dependence and vulnerability to specific targeted therapies. These models have been used to estimate oncogenicity of driver mutations or efficacies of molecularly targeted drugs. In addition, Ba/F3 models, together with N‐ethyl‐N‐nitrosourea mutagenesis, have been used to derive acquired resistant cells to investigate on‐target resistance mechanisms. Here, we reviewed studies that used Ba/F3 models with *EGFR* mutations, *ALK/ROS1/NTRK/RET* fusions, *MET* exon 14 skipping mutations, or *KRAS* G12C mutations to investigate secondary/tertiary drug resistant mutations. We determined that 68% of resistance mutations reproducibly detected in clinical cases were also found in Ba/F3 models. In addition, sensitivity data generated with Ba/F3 models correlated well with clinical responses to each drug. Ba/F3 models are useful to comprehensively identify potential mutations that induce resistance to molecularly targeted drugs and to explore drugs to overcome the resistance.

## Graphical abstract

In this review paper, we comprehensively reviewed published studies that used the Ba/F3 model as a tool to investigate resistant mutations to molecular targeted drugs in non‐small‐cell lung cancers (NSCLCs) with driver mutations. Through a comparison with resistant mutations detected in clinical specimens, we found that 68% of them were also identified in Ba/F3 models. The Ba/F3 model is useful in exploring and/or validating on‐target acquired resistance mechanisms to molecular targeted therapies in NSCLCs.

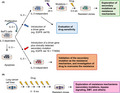

**Abbreviations**
1G/2G/3G: first/second/third generation
ALK: anaplastic lymphoma kinase
BRAF: v‐raf murine sarcoma viral oncogene homolog B1
EGFR: epidermal growth factor receptor
ENU: N‐ethyl‐N‐nitrosourea
IL‐3: interleukin‐3
KRAS: v‐Ki‐ras2 Kirsten rat sarcoma viral oncogene homolog
MET: mesenchymal‐epithelial transition factor
NSCLC: non‐small‐cell lung cancer
NTRK: neurotrophic tropomyosin receptor kinase
RET: rearranged during transfection
ROS1: c‐ros oncogene 1
RTK: receptor tyrosine kinase
SCLC: small‐cell lung cancer
TKI: tyrosine kinase inhibitor
TRKA/B/C: tropomyosin receptor kinase

## 1 INTRODUCTION

For unresectable/advanced NSCLC, molecular targeted therapies are the standard, front‐line treatment for NSCLCs that harbor one of the following driver gene alterations: *EGFR* mutations, *ALK* fusions, *ROS1* fusions, *RET* fusions, *BRAF* V600E mutation, *MET* exon 14 skipping mutation, and *NTRK* fusions. In addition, sotorasib (KRAS G12C inhibitor), amivantamab‐vmjw (anti‐EGFR/MET bispecific Ab), and mobocertinib have recently joined the list of FDA‐approved drugs, and inhibitors targeting HER2 have been investigated with promising outcomes in early phase clinical trials^1^ (Table S1).

Despite the initial dramatic response to these molecular targeted drugs, the emergence of acquired resistance is inevitable. Molecular mechanisms of acquired resistance can be classified into three categories: (i) on‐target alterations such as secondary mutations, (ii) activation of accessory or downstream pathways, and (iii) phenotypic transformation, such as the epithelial‐mesenchymal transition or SCLC transformation.^2^ Identification of acquired resistance mechanisms potentially leads to mechanism‐oriented, second line treatments with promising efficacies.^3^, ^4^

## 2 IN VITRO MODELS FOR RESISTANCE MECHANISM ANALYSES

**FIGURE 1:**
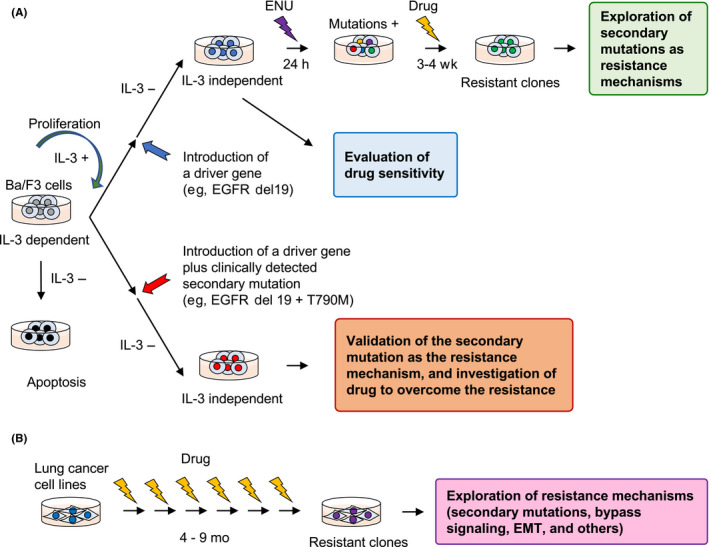
Ba/F3 model and lung cancer cell lines as tools for mechanistic analysis of resistance to molecular targeted drugs. A, Parental Ba/F3 cells are interleukin‐3 (IL‐3)‐dependent; however, they transform to IL‐3‐independent when a driver mutation is introduced. This model is extremely sensitive to molecular targeted drugs that can inhibit the introduced driver mutation. N‐ethyl‐N‐nitrosourea (ENU) mutagenesis can cause various secondary mutations in the introduced driver gene, and short‐term treatment with a molecular targeted drug will select Ba/F3 clones with drug‐resistant mutations. Ba/F3 cells are also used as a validation tool for secondary mutations identified in clinical samples. Ba/F3 cells harboring a driver mutation plus a secondary mutation are used to evaluate drug sensitivity or investigate drugs that can overcome the initial drug resistance. B, Commercially available, conventional lung cancer cell lines or patient‐derived lung cancer cells are used to establish models to study acquired resistance to molecular targeted drugs. Cell lines are exposed to the drug for at least 3‐4 mo until these cells become resistant to the drug. EGFR, epidermal growth factor receptor; EMT, epithelial‐mesenchymal transition

In vitro models have played important roles in elucidating resistance mechanisms that develop after treatment with molecular targeted drugs. These in vitro models can be classified into three groups: (i) conventional cell lines established from lung cancer patients a long time ago, many of which were established by Professors Adi F. Gazdar and John Minna approximately 30 years ago,^5^ (ii) newly derived cell lines and tumor organoids from patients, and (iii) Ba/F3 models, which are the focus of this review article (Figure 1A).

After discoveries of *EGFR* mutations and *ALK* fusions in NSCLCs, many researchers have used conventional cell lines with either *EGFR* mutations or *ALK* fusions to explore mechanisms of acquired resistance to the respective TKIs (Figure 1B).^6^, ^7^, ^8^ These studies identified numerous mechanisms, as listed above, and revealed that secondary mutations were the most common mechanism of acquired resistance.^2^ Following the *EGFR* mutations and *ALK* fusions, several other driver mutations, such as *BRAF V600E* mutation, *ROS1* fusions, *NTRK* fusions, and *MET* exon 14 skipping mutations, were discovered in NSCLCs, and molecular targeted drugs for these genetic alterations have been developed. However, due to the rarity of the latter driver mutations, lung cancer‐derived cell lines that harbor one of these mutations are usually unavailable. Therefore, Ba/F3 cells that have been transduced with these mutated driver genes are an important tool for mechanistic and therapeutic investigations.

## 3 BASICS OF BA/F3 CELLS AS A TOOL TO GENERATE ONCOGENE‐DEPENDENT CELL LINE MODELS

Ba/F3 is a murine, IL‐3‐dependent, pro‐B cell line, which is a popular system that can resolve the limited availability of lung cancer patient‐derived cells with rare driver mutations. The origin of Ba/F3 cells is somewhat unclear because they were initially reported as IL‐3‐dependent pro‐B cells isolated from the bone marrow of Balb/c mice.^9^ However, single nucleotide polymorphism genotyping revealed that this cell line was derived from C3H mice.^10^ Nevertheless, Ba/F3 cells have served as an important tool for oncology research because the removal of IL‐3 causes loss of viability. Ba/F3 cells can grow in the presence of 5 ng/mL IL‐3 with a doubling time of 8 hours.^11^ Introduction of a driver gene mutation can render Ba/F3 cells independent of IL‐3 but dependent on the introduced driver gene. Therefore, this simple oncogene dependency creates a straightforward tool for testing the sensitivity of Ba/F3 cells to molecular targeted drugs (Figure 1A). Ba/F3 cells have been used to investigate the transforming ability of driver oncogenes since Daley and Baltimore reported in 1988 that the introduction of *BCR/ABL* produced IL‐3‐independent growth.^12^ Using a mutagenesis PCR technique, Ba/F3 cells can be generated with any driver mutation that is found in NSCLCs.

However, it should be noted that Ba/F3 models have some limitations that should be considered when we evaluate the results obtained from Ba/F3 experiments. First, it is usually difficult to control the expression level (as well as the introduced gene copy number) of the transfected driver gene. Second, because only a single driver mutation is usually introduced into Ba/F3 cells, the established Ba/F3 clone does not carry the WT allele of the driver gene. Third, because Ba/F3 cells do not have innate human genes, it is impossible to evaluate the impacts of heterodimers between introduced oncogenes and other RTKs (for example, EGFR is reported to form heterodimers with other ERBB members such as ERBB3^13^). However, it should be mentioned that the requirement of homodimerization can be evaluable using Ba/F3 models; for example, using NIH‐3T3 cells and Ba/F3 cells, a previous study reported that EGFR L858R mutant required homodimerization for activation but EGFR exon 19 deletion, exon 20 insertion, and L858R/T790M did not require homodimerization^14^.

## 4 BA/F3 CELLS AS A TOOL TO IDENTIFY ON‐TARGET ACQUIRED RESISTANCE MECHANISMS

Exposure of transfected Ba/F3 cells to increasing concentrations of molecular targeted drugs will often result in the development of drug resistance. The use of ENU can facilitate and shorten the process of resistance induction (Figure 1A). However, it is difficult to identify acquired resistance mechanisms other than secondary mutations using the Ba/F3 model. One of the first applications of Ba/F3 cells for identifying secondary resistance mutations was reported by Ercan et al who used ENU mutagenesis and identified an EGFR C797S mutation as a mechanism of osimertinib resistance.^15^ This study was followed by the identification of a C797S mutation in a patient who developed acquired resistance to osimertinib.^16^ Furthermore, Katayama et al used Ba/F3 cells to identify secondary *ROS1* mutations that could cause crizotinib or ceritinib resistance.^17^

**FIGURE 2:**
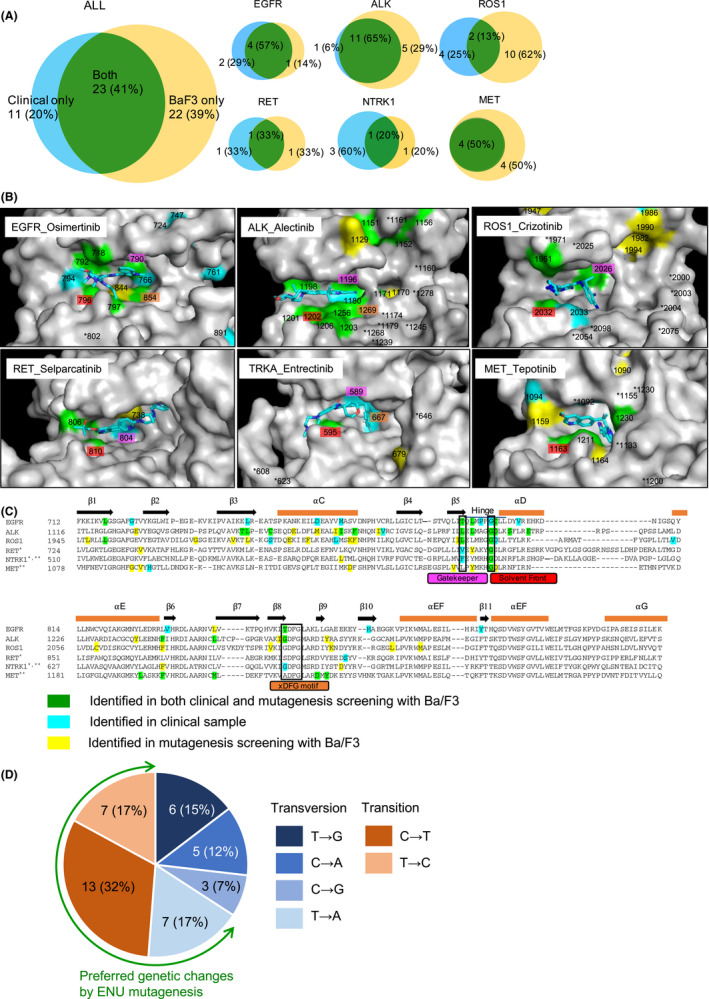
Correlations between resistance mutations identified in clinical specimens and those found in Ba/F3 models. A, The Venn diagrams indicate the numbers of residues in which resistance mutations were reproducibly identified in clinical specimens from non‐small‐cell lung cancer patients and/or Ba/F3 models. B, Structural models of the receptor tyrosine kinase (RTK) drug binding pocket and one of the molecular targeted drugs for each RTK. The residues in which resistance mutations were identified in both patients and Ba/F3 models, only in patients, and only in Ba/F3 models are colored in green, blue, and yellow, respectively. Gatekeeper residues, solvent front residues, and the “x” residue of xDFG motif are colored in pink, red, and orange, respectively. C, Locations of residues in which resistance mutations were identified either in patients or in Ba/F3 models are summarized. The color codes are identical to those described in Figure 2B. The residues described here but not in Figure 2C are not located in the surface of the protein or not located in the drug binding area. D, Patterns of base substitutions identified in our recent studies^19^, ^20^, ^21^ that used Ba/F3 models and N‐ethyl‐N‐nitrosourea (ENU) mutagenesis. Secondary mutation data are from following references:: *EGFR* mutation^6^, ^16^, ^28^, ^29^, ^30^, ^32^, ^36^, ^39^, ^40^, ^41^, ^42^, ^43^, ^97^, ^98^, ^99^, ^100^, ^101^, ^102^, ^103^, ^104^, ^105^, ^106^, ^107^, ^108^, ^109^, *ALK* fusion^45^, ^46^, ^47^, ^48^, ^49^, ^51^, ^53^, ^54^, ^55^, ^56^, ^57^, ^110^, ^111^, ^112^, ^113^, ^114^, ^115^, ^116^, ^117^, *ROS1* fusion^17^, ^53^, ^59^, ^63^, ^64^, ^118^, ^119^, ^120^, ^121^, ^122^, ^123^, *RET* fusion^69^, ^70^, ^124^, ^125^, ^126^, *NTRK* fusion^73^, ^75^, ^76^, ^127^, and *MET* exon 14 skipping^20^, ^21^, ^81^, ^83^, ^84^, ^85^, ^86^, ^88^, ^89^, ^95^, ^132^. *In *RET* and *NTRK* fusions, the resistance mutations that emerged in other type of cancers are also included. **In *NTRK* fusion and *MET* exon 14 skipping mutation, the resistance mutations that emerged against unapproved drugs are also included. Protein Database IDs: EGFR_osimertinib, 6JWL; ALK_alectinib, 3AOX; ROS1_crizotinib, 3ZBF; RET_selparcatinib, 7JU6; TRKA_entrectinib, 5KVT; MET_tepotinib, 4XMO

Secondary mutations identified in Ba/F3 models and clinical specimens are not always identical. We classified secondary resistance mutations into three groups: (i) those found in both clinical specimens and Ba/F3 models, (ii) those found only in clinical specimens, and (iii) those found only in Ba/F3 models (Figure 2A‐C). Thirty‐four amino acid residues in EGFR, ALK, ROS1, RET, NTRK1, and MET proteins contained secondary/tertiary mutations and were reproducibly identified in clinical samples obtained from NSCLCs (and other type of cancers for RET/NTRK fusions). Of these 34 residues, 23 (68%) of these mutations were also identified in Ba/F3 models (Figure 2A). However, mutations in 22 other residues have been reported only in Ba/F3 models. We noted that the data on *ROS1*, *NTRK*, or *MET* mutations were the primary cause of discordance, which was likely because of the rarity of clinical reports that examined resistance to these driver mutations. Therefore, the discordant data are expected to decrease as more samples are analyzed in the future.

N‐ethyl‐N‐nitrosourea mutagenesis preferentially induces T→C or C→T transitions and T→A transversions,^18^ which is a limitation using the Ba/F3 model. The frequencies of these genetic changes were calculated using data from our recent publications.^19^, ^20^, ^21^ We found that the preferential changes were more frequent (66%) than other genetic changes (Figure 2D). In addition, as described above, Ba/F3 clone does not carry the WT allele of the introduced driver mutation. Therefore, the secondary mutation always occurs *in cis* with the activating mutation. Secondary mutation *in cis* is frequent in clinic,^22^ however, there are some reports that describe the occurrence of *in trans* secondary mutations.^23^, ^24^

## 5 BA/F3 CELLS AS A TOOL TO EXPLORE NOVEL AGENTS TO OVERCOME ON‐TARGET RESISTANCE

The Ba/F3 cell model is also useful to examine the roles of secondary mutations with unknown significance that are found in TKI‐refractory patient specimens. Ba/F3 cell lines can be produced with any driver or secondary (or tertiary) mutation (Figure 1A) and used to evaluate the efficacy of drugs. To our knowledge, in the field of lung cancer research, Ba/F3 cells were first used for this purpose, that is, to confirm that the EGFR T790M secondary mutation conferred acquired resistance to gefitinib, a 1G‐EGFR‐TKI.^25^

**FIGURE 3:**
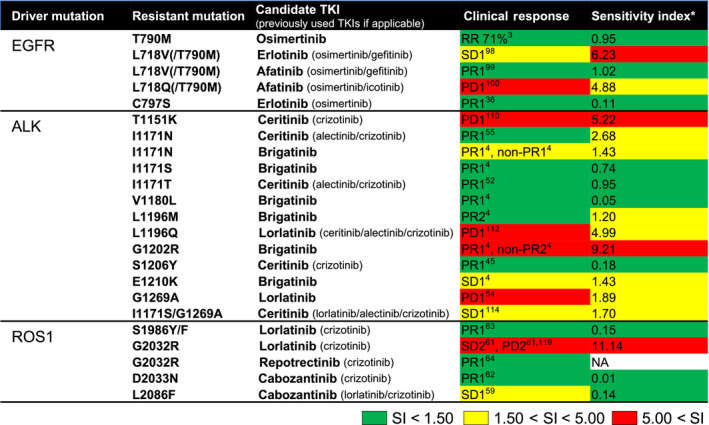
Correlations between clinical efficacy of tyrosine kinase inhibitors (TKIs) and sensitivity index using Ba/F3 cells. Clinical efficacies of EGFR, ALK, or ROS1‐TKIs in anecdotal cases with secondary or tertiary mutations are summarized. Patient data without RECIST were not included. For secondary mutations with inconsistent clinical responses, the color code was based on the responses of all patients and determined after discussion among the authors. *Sensitivity index (SI) values for Ba/F3 cells (IC_50_ values × 100/C_trough_ in clinical trials) with the respective secondary or tertiary mutations are summarized to show the correlations between clinical efficacy and data generated with Ba/F3 models. The measured SI values were color coded as follows: ≤1.50, green; 1.50–5.00, yellow; and >5.00, red. NA, not available; PD, progressive disease; PR, partial response; RR, response rate; SD, stable disease

Ba/F3 cells with secondary mutations can be used to explore novel TKIs that can overcome drug resistance. These types of studies have enabled the development of catalogues that summarize the correlations between secondary mutations and TKI efficacies (Tables S2‐S4). The clinical utility of these catalogues is presented in Figure 3 for some anecdotal cases. We summarized sensitivity indices (IC_50_ values adjusted with clinically achievable concentrations of each TKI) generated from Ba/F3 cell experiments and clinical responses in NSCLC patients for each secondary/tertiary mutation together with the *EGFR*, *ALK*, and *ROS1* driver mutation (Figure 3). Sensitivity indices correlated well with clinical responses, further signifying the importance of Ba/F3 data for predicting drug efficacies in patients who have acquired secondary/tertiary mutations.

## 6 EXPLORATION OF SECONDARY/TERTIARY MUTATIONS THAT CAUSE RESISTANCE TO EACH KINASE INHIBITOR USING THE BA/F3 SYSTEM

### 6.1 Shared structures between RTKs

Several important structural sites or motifs are shared among RTKs and include the gatekeeper site, the solvent‐front site, and the xDFG (Asp‐Phe‐Gly) motif (Figure 2B,C). The gatekeeper site is in the innermost part of the ATP‐binding pocket, and this single amino acid determines the shape of the hydrophobic pocket. A secondary mutation at this site will cause TKI resistance by sterically blocking the binding of TKIs and/or by increasing ATP affinity and reducing the potency of ATP‐competitive TKIs. Epidermal growth factor receptor T790M (the most frequent secondary mutation after 1G or 2G EGFR‐TKI treatment) and ALK L1196M are two well‐known gatekeeper mutations.

The solvent front is a hydrophilic amino acid (often glycine) at the entrance of the ATP binding pocket, by which multiple TKIs must pass to enter the pocket. Therefore, structural changes at this site will inhibit TKI binding. Secondary mutations (often glycine to arginine) at this position occur frequently in fusion gene‐derived driver proteins, such as those involving ALK, ROS1, and NTRK, and result in narrowing of the entrance.

The xDFG motif, which is the initiation point of the activation segment of RTKs, adopts an “in” conformation in catalytically active kinases, where the motif is flipped outward at kinase inactivation. Although the xDFG motif is well conserved, secondary mutations at the Asp‐Phe‐Gly site have not been reported in either Ba/F3 models or clinical specimens. Some secondary mutations have been reported at the “x” position in clinical samples and/or Ba/F3 experiments in *EGFR*‐, *ALK*‐, and *NTRK*‐driven NSCLCs. Considering the homology between RTKs is sometimes helpful to understand resistance mutations and explore effective TKIs that might overcome drug resistance.

### 6.2 *EGFR* mutations

#### 6.2.1 *EGFR* secondary mutations that confer resistance to 1G or 2G EGFR‐TKIs

*EGFR* mutations are one of the most frequent driver mutations in lung adenocarcinomas and are present in approximately 17% of Caucasians^26^ and 40% of East Asian^27^ patients. In clinical practice, the secondary T790M (gatekeeper) mutation is the most frequent mechanism (~50%) of acquired resistance to 1G or 2G EGFR‐TKIs, although very rare secondary mutations, such as L747S,^28^ D761Y,^29^ or T854A (xDFG motif),^30^ have also been reported.^7^, ^31^ Similar to clinical observations, several groups have reported the emergence of the T790M secondary mutation in Ba/F3 models after 1G or 2G EGFR‐TKI treatment.^19^, ^32^ In addition, emergence of rare secondary mutations, such as C797S (afatinib/dacomitinib),^19^ L792H/F (afatinib),^19^, ^32^ or T854A (afatinib),^19^ have been reported in Ba/F3 models (Figure 2B,C).

#### 6.2.2 *EGFR* secondary/tertiary mutations that confer resistance to osimertinib

Osimertinib, a 3G irreversible EGFR‐TKI, is used either as a front‐line treatment or a second‐line treatment if 1G or 2G EGFR‐TKI therapy fails because of the development of a T790M secondary mutation. In the front‐line setting, secondary *EGFR* mutation, including C797S, L718Q, G724S, or S768I, were identified in only 6%–10% of plasma samples obtained from NSCLC patients after disease progression, while bypass pathway activation or SCLC transformation were more common.^33^, ^34^ L718Q and L718V mutations were also identified in tissue biopsy samples after acquisition of resistance to front‐line osimertinib treatment.^35^ In Ba/F3 cells, C797S was the only secondary mutation that was identified after first‐line osimertinib treatment model thus far.^32^ We and others have observed that 1G EGFR‐TKIs are active against the C797S mutated cells, which has been confirmed in the clinical setting.^36^

After second‐ or later‐line osimertinib treatment of lung cancer patients with secondary T790M mutation, the acquisition of tertiary mutations is relatively frequent (10%–26%).^34^, ^37^ Tertiary mutations found in clinical samples included L718Q, M766Q, L792X, G796X (solvent front), C797X, and exon 20 insertion mutations (Figure 2B,C).^38^, ^39^, ^40^, ^41^, ^42^ Ba/F3 cells were widely used to validate the roles of these tertiary mutations (Figure 1A).^41^, ^43^

Table S2 summarizes the IC_50_ values of erlotinib, gefitinib, afatinib, dacomitinib, osimertinib, and brigatinib in Ba/F3 cells with secondary/tertiary *EGFR* gene mutations. In addition, the mutations identified in EGFR‐TKI refractory patients and/or Ba/F3 models are illustrated in Figure 2B,C.

### 6.3 *ALK* secondary/tertiary mutations

*ALK* fusions are identified in approximately 3%–4% of NSCLC patients with a prevalence in young never‐smokers with adenocarcinoma.^44^ Several ALK‐targeting TKIs, including crizotinib (1G), alectinib (2G), ceritinib (2G), brigatinib (2G), and lorlatinib (3G), are currently used in clinical practice. Because crizotinib was the first ALK‐TKI developed, many of the reports regarding acquired resistance mutations after ALK‐TKI treatment are for crizotinib or sequential treatment with 2G or 3G TKIs after initial crizotinib therapy.

In a large systematic analysis of resistance mechanisms to crizotinib and 2G ALK TKIs, secondary mutations were identified in 20% (11/55) of crizotinib, 54% (13/24) of ceritinib, and 53% (9/17) of alectinib refractory tumors.^45^ As shown in Figure 2B,C, various secondary mutations have been reported in clinical samples after crizotinib treatment, including the first reported L1196M (gatekeeper) and C1156Y mutations^46^ and L1151Tins, L1152R, G1202R (solvent front), S1206Y, and G1269A (xDFG motif) mutations that followed.^47^, ^48^, ^49^ The G1202R solvent front mutation causes resistance to both of alectinib and ceritinib, in addition, I1171N/S/T or F1174C/L mutations were reported to cause alectinib resistance or ceritinib resistance, respectively.^45^, ^50^, ^51^, ^52^, ^53^, ^54^, ^55^ Ba/F3 models were frequently used to confirm these clinical findings.^45^, ^51^, ^52^, ^54^

**FIGURE 4:**
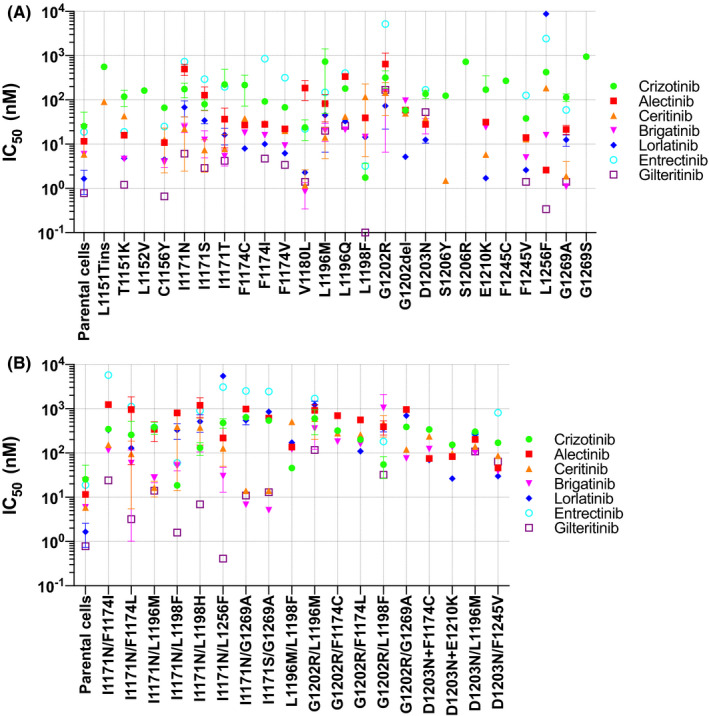
The IC_50_ values of Ba/F3 cells harboring the *EML4/ALK* fusion plus resistance mutations for each anaplastic lymphoma kinase (ALK)‐tyrosine kinase inhibitor (TKI). IC_50_ values for each ALK‐TKI in Ba/F3 cells harboring the *EML4/ALK* fusion gene with secondary/tertiary mutations. Each plot indicates the average value of the IC_50_ described in each manuscript reviewed

Lorlatinib, a 3G TKI, is active against the majority of secondary mutations that could cause resistance to 1G or 2G TKIs, including G1202R.^45^, ^56^ However, clinical use of lorlatinib after treatment failure of 1G and/or 2G ALK‐TKIs, resulted in the emergence of tertiary mutations.^45^, ^54^, ^56^ Among these tertiary mutations, ALK L1198F was detected in a patient who developed acquired resistance to lorlatinib after previously developing a secondary C1156Y mutation against front‐line crizotinib. Interestingly, the lorlatinib‐resistant tumor (EML4‐ALK/C1156Y/L1198F) responded to crizotinib again.^57^ In vitro experiments using Ba/F3 cells supported this clinical phenomenon; L1198F mutant and C1156Y/L1198F mutant cells were both sensitive to crizotinib but C1156Y mutant cells were not.^45^, ^57^ In addition, ENU mutagenesis screening of Ba/F3 cells identified clinically meaningful tertiary mutations; for example, L1196M/G1202R mutations established in Ba/F3 models were also identified in patients who received lorlatinib or brigatinib after crizotinib treatment failure.^56^, ^58^ Mutations identified in ALK‐TKI refractory patients and/or Ba/F3 models are illustrated in Figure 2B,C. We summarized the IC_50_ data for ALK‐TKIs using Ba/F3 cells with secondary or tertiary mutations in Figure 4 (detailed IC_50_ values are presented in Table S3).

### 6.4 *ROS1* secondary/tertiary mutations

*ROS1* fusions are found in 1%–2% of NSCLC patients and occur preferentially in young lung adenocarcinoma patients without a smoking history. Crizotinib, entrectinib, ceritinib, and lorlatinib are currently available for NSCLC patients with *ROS1* fusions in the United States. As observed for ALK rearrangement, the majority of reported data on acquired resistance mechanisms in *ROS1*‐positive NSCLC patients are for crizotinib treatment. A case series reported that secondary *ROS1* mutations were detected in 38% (16/42)‐53% (9/16) of crizotinib‐resistant specimens,^59^, ^60^ and G2032R (solvent front), D2033N, S1986F, and L2026M (gatekeeper) mutations were the exact secondary mutations.^59^, ^61^

Ba/F3 models have also been used to identify secondary mutations that may confer resistance to ROS1‐TKIs. Several groups have carried out ENU mutagenesis screening with crizotinib and ceritinib in Ba/F3 cells containing a *CD74‐ROS1* fusion and identified several secondary mutations, including G2032R, D2033N, and L2026M (Figure 2B,C).^17^, ^62^ Furthermore, these studies showed that the D2033N, but not G2032R, mutation could be overcome by lorlatinib treatment (Table S4). *ROS1* mutations have been identified in 46% (13/28) of lorlatinib/crizotinib‐resistant patients and include G2032R, L2086F, G2032R/L2086F, S2032R/L2086F/S1986F, and S1986F/L2000V mutations.^59^

Based on the homology between *ROS1* and *ALK* kinase domains, several groups proposed that certain TKIs may overcome *ROS1* secondary mutations and confirmed their hypothesis using Ba/F3 models. For example, the *ROS1* S1986Y/F is homologous to the *ALK* C1156 mutation, which is sensitive to lorlatinib, and lorlatinib overcomes crizotinib/ceritinib‐resistance conferred by ROS1 S1986Y/F mutations.^63^ The *ROS1* L2026M crizotinib‐resistant mutation is located at the gatekeeper position (homologous to *ALK* L1196M), and Ba/F3 cells with a *CD74‐ROS1* fusion plus L2026M mutation are sensitive to ceritinib, which is similar to Ba/F3 cells with an *EML4‐ALK* fusion plus L1196M (gatekeeper) mutation.^51^ In addition to these ROS1/ALK TKIs, experiments using Ba/F3 models have shown that repotrectinib (a ROS1/TRKA‐C/ALK inhibitor),^64^, ^65^ DS‐6051b (next generation ROS1/NTRK inhibitor),^66^ or cabozantinib (a multikinase TKI)^17^, ^62^, ^67^ have potent activity against crizotinib‐resistant cells with *ROS1* mutations, including G2032R. The IC_50_ values of ROS1‐TKIs in Ba/F3 cells with secondary/tertiary mutations are summarized in Table S4.

### 6.5 *RET* secondary mutations

*RET* fusions are rare driver mutations that are present in less than 0.9% of NSCLCs.^68^ The RET‐specific TKIs selpercatinib and pralsetinib have been approved in the United States, and the former was recently approved in Japan (Table S1). Because of the rarity of *RET* fusions in NSCLCs, the incidence of secondary mutations resulting in acquired resistance to RET‐TKIs is currently unclear. In the analyses of selpercatinib‐ or pralsetinib‐resistant patients with RET fusions (NSCLC or medullary thyroid cancer), several secondary mutations have been reported (Figure 2B,C). Acquired G810R/S/C/V solvent front mutations were detected by plasma cell‐free tumor DNA analysis in an NSCLC patient with a *KIF5B‐RET* fusion who progressed after selpercatinib treatment.^69^ The *RET* G810C secondary mutation was also identified in an NSCLC patient with a *CCDC6‐RET* fusion who acquired resistance to selpercatinib, and this finding was supported by Ba/F3 cell experiments. The IC_50_ values for selpercatinib or pralsetinib in Ba/F3 cells harboring *KIF5B‐RET* plus G810S/C/R were 42‐ to 334‐fold higher than Ba/F3 cells with only the *KIF5B‐RET* fusion.^70^ TPX‐0046, a next‐generation RET/SRC inhibitor, showed a much lower IC_50_ value than selpercatinib in G810R‐positive Ba/F3 cells. The phase I/II clinical trial investigating the use of TPX‐0046 for *RET*‐altered NSCLC and medullary thyroid cancer is currently ongoing (NCT04161391).

### 6.6 *NTRK* secondary mutations

*NTRK* includes *NTRK1*, *NTRK2*, and *NTRK3* that encode TRKA, TRKB, and TRKC proteins, respectively. *NTRK* fusions are detected in various type of cancers, including secretory breast carcinoma, mammary analogue secretary carcinoma, congenital mesoblastic nephroma, and infantile fibrosarcoma.^71^ In NSCLC, the frequency of *NTRK* fusions is reported to be less than 1%.^72^ In phase I/II trials of solid tumors harboring *NTRK* fusions, including NSCLCs, both larotrectinib and entrectinib showed significant responses (Table S1).^73^, ^74^

As resistant mechanisms, *NTRK1* G595R (solvent front) and G667S (xDFG motif) mutations were detected in a *TPR‐NTRK1* fusion‐positive lung cancer patient who acquired resistance to larotrectinib (Figure 2B,C).^73^ Ba/F3 models harboring a *TPM3‐NTRK1* fusion plus G667C or G595R mutation were used to explore TKIs that can overcome these secondary mutations. Nintedanib, ponatinib, cabozantinib, and foretinib were active against cells with the G667C mutation but inactive against cells with the G595R mutation.^75^ Selitrectinib (LOXO‐195), TPX‐0005, and ONO5390556 have shown potent activity in preclinical models of *NRTK1* G595R or G667C mutations.^71^, ^76^ In a clinical trial, selitrectinib showed a 45% (9/20) objective response rate in TRK fusion‐positive patients with solid tumors who had been treated with more than one TRK inhibitor.^77^

### 6.7 *MET* secondary mutations

The *MET* exon 14 skipping mutation is a driver mutation detectable in approximately 4% and 20% of patients with lung adenocarcinoma and pleomorphic carcinoma, respectively.^78^, ^79^ Several types of MET‐TKIs have been developed: type I inhibitors (crizotinib, capmatinib, tepotinib, savolitinib) that bind the active form of MET, and type II inhibitors (merestinib, glesatinib, cabozantinib) that bind the inactive form of MET.^80^ Among these MET‐TKIs, tepotinib and capmatinib have been approved for clinical use in the United States and Japan.

In the analysis of 20 patients who were treated with MET‐TKIs, on‐target and off‐target resistance was identified in 35% and 45% of patients, respectively.^81^ Among patients with on‐target acquired resistance to crizotinib, various secondary mutations were identified, including G1163R (solvent front), L1195V, F1200I, D1228N/H/A, and Y1230C/. ^81^, ^82^, ^83^, ^84^, ^85^, ^86^ It is noteworthy that several secondary mutations can emerge simultaneously after crizotinib treatment. For example, two NSCLC patients each developed four missense mutations simultaneously after crizotinib treatment: (i) G1163R, D1228H, D1228A, and Y1230H, and (ii) G1163R, D1228N, Y1230H, and 1230S.^85^, ^87^ In NSCLC patients with acquired resistance to capmatinib, D1228N/Y mutations have been repeatedly reported.^81^, ^88^

Using a Ba/F3 model with *MET* exon 14 skipping, we comprehensively examined secondary mutations that could cause MET‐TKI resistance using various type I and II MET‐TKIs.^20^ D1228 and Y1230 mutations frequently occurred after type I MET‐TKI exposure, and L1195 and F1200 mutations tended to emerge after type II MET‐TKI treatment. Therefore, from Ba/F3 experiments and clinical observations, it is reasonable to suggest that sequential use of type II MET‐TKIs might overcome secondary mutations caused by type I MET‐TKIs and vice versa.^20^, ^89^

### 6.8 *KRAS* secondary mutations

*KRAS* mutations are present in approximately 15%–25% of NSCLC patients.^90^, ^91^ Recently, two covalent inhibitors, sotorasib and adagrasib, have shown potent clinical activity against cells with the *KRAS* G12C mutation, which accounts for approximately 40% of all *KRAS* mutations in NSCLCs.^92^, ^93^, ^94^ Sotorasib was approved for clinical use in the United States in May 2021 (Table S1).

To identify mechanisms of on‐target resistance to *KRAS* G12C inhibitors, we undertook ENU mutagenesis using the Ba/F3 model.^21^ *KRAS* Y96D/S mutations induced acquired resistance to both sotorasib and adagrasib.^21^ Other *KRAS* secondary mutations, such as G13D, A59S/T, Q61L, and R68M/S were also detected. A *KRAS* Y96D mutation was also detected in a liquid biopsy of an NSCLC patient who acquired resistance to adagrasib, which was validated as the refractory mutation using the Ba/F3 model.^95^ Furthermore, acquired *KRAS* mutations after adagrasib monotherapy, including G12D/R/V/W, G13D, Q61H, R68S, H95D/Q/R, and Y96C, were detected in the analysis of 27 patients with NSCLC, 10 with colorectal cancer, and one with appendiceal cancer who achieved tumor reduction, in addition to *EGFR* or *MET* amplification and other MAPK kinase gene mutations.^96^ The Ba/F3 model was used in this study to comprehensively validate the sensitivity of *KRAS* mutations to KRAS G12C inhibitors.^96^

## 7 CONCLUSIONS

The development and approvals of targeted drugs have improved treatment outcomes of patients with NSCLC harboring driver mutations. This progress in oncology is encouraging; however, mechanistic analyses of acquired resistance to these targeted drugs is necessary to further improve patient outcomes. As described in this review, the Ba/F3 cell model is useful to validate the oncogenic roles of these mutations. Furthermore, exploratory studies using Ba/F3 cells with ENU mutagenesis will be beneficial to comprehensively detect mutations that could promote resistance to targeted drugs.

## DISCLOSURE

T. Mitsudomi serves as an editorial board member of *Cancer Science*. T. Mitsudomi has received research funding from Boehringer Ingelheim, Apollomics Icon Japan, Covance, and Clinipace, research grants from Boehringer Ingelheim, Chugai, Ono, Pfizer, and Covidien, and lecture fees from AstraZeneca, Boehringer Ingelheim, Chugai, Eli‐Lilly, Pfizer, and Merck Sharp and Dohme. The other authors have no conflict of interest.

## Supporting information

Appendix S1
