# Supplementary material for: Utility of the Ba/F3 cell system for exploring on‐target mechanisms of resistance to targeted therapies for lung cancer
Source: Cancer Sci. 2022 Jan 23;113(3):815–27. doi: 10.1111/cas.15263 (PMC8898722; doi:10.1111/cas.15263)
Supplement: Supplementary file 1 — Appendix S1 [file CAS-113-815-s001.pdf]

Supplemental Tables

Table S1. List of PMDA and FDA approved targeted therapy drugs for driver oncogenes in NSCLC

Target	Drug	PMDA (Jpn)*1	FDA (US)*2
EGFR	Gefitinib	○	○
	Erlotinib	○	○
	Afatinib	○	○
	Dacomitinib	○	○
	Osimertinib	○	○
	Amivantamab-vmjw		○
	Mobocertinib		○
ALK	Crizotinib	○	○
	Alectinib	○	○
	Ceritinib	○	○
	Lorlatinib	○	○
	Brigatinib	○	○
ROS1	Crizotinib	○	○
	Entrectinib	○	○
	Ceritinib		○
	Lorlatinib		○
NTRK	Entrectinib	○	○
	Larotrectinib	○	○
RET	Selpercatinib	○	○
	Pralsetinib		○
MET exon 14 skipping	Capmatinib	○	○
	Tepotinib	○	○
BRAF V600E	Dabrafenib plus Trametinib	○	○
KRAS G12C	Sotorasib		○

*1 Pharmaceuticals and Medical Devices Agency; https://www.pmda.go.jp/english/index.html
*2 NIH Drugs Approved for Lung Cacner; https://www.cancer.gov/about-cancer/treatment/drugs/lung

Table S2. The summery of IC50 values of Ba/F3 cells with EGFR del19/L858R plus resistance mutations to each EGFR-TKIs

[nM]

EGFR Ba/F3		Erlotonib	Gefitinib	Afatinib	Dacomitinib	Osimertinib	Brigatinib	References*
Del19		1.2	4.13	0.047	0.12	0.78~0.91	17.6	S1, S2
	L718V	14.79		0.464	0.295	10.57	322.1	S1
	T790M/L718Q		>1000			>500		S2
	G724S	1.93		0.025	0.025	3.79	55.7	S1
	T790M	6686	>1000	144	271	0.7~3.48	289.9	S1, S2
	L792F	3.31	3.89	0.3	0.19	4.01~4.81	155.1	S1, S2
	T790M/L792F		>1000			10.04		S2
	L792H	11.61		0.57	0.073	11.95	140.7	S1
	T790M/L792H		>1000			97.49		S2
	L792Y		2.85			36.64		S2
	T790M/L792Y		>1000			33.04		S2
	C797S	3.33	3.95	4.78	5.88	925.4~>1000	70.2	S1
	T790M/C797S		>1000			>1000		S2
	C797S/T854A		43.8	26.4		656.8	14.8	S3
	T854A		55.8	0.6		1.7	11.1	S3
L858R		9.37	6.65	0.616	0.12	2.34~3.55	277.2	S1, S2
	L718Q	424.6	<500	3.37	1.99	533.0~>1000	629.4	S1, S2
	T790M/L718Q		>1000			>1000		S2
	L718V	184.9		0.703	0.229	167.9	793.9	S1
	L747S	8.68		0.24	0.41	1.74		S4
	D761Y	11.1		0.82	1.16	2.74		S4
	L766Q	4450		46.4		50.62		S5
	T790M	5596	>1000	99.52	94.177	2.8~4.77	335.1	S1, S2
	L792F	14.87	10.2	0.97	0.21	29.53~36.29	556.8	S1, S2
	T790M/L792F		>1000			38.65		S2
	L792H	28.15	31.25	1.25	0.16	44.71~72.41	662.3	S1, S2
	T790M/L792H	>2000	>1000	1112		54.75~222		S2, S6
	T790M/L792Y		>1000			49.57		S2
	T790M/G796R	>2000		569		992		S6
	C797G	1.26		1.38	2.18	561.21	58.31	S1
	C797S	4.81	9.19	6.68	4.63	918.0~>1000	174.3	S1, S2
	T790M/C797S		>1000			>1000		S2
	C797S/T854A		101.8	60.6		1007	72.3	S3
	T854A	85.9	66.3	0.5-2.13	3.19	1.98-2.3	31.8	S3, S4
	L861Q		169.1	1.8		28.8		S7

We searched in PubMed with the keywords "EGFR", "lung cancer", "resistance", "Ba/F3" on March 3, 2021. Title and abstract of these manuscripts were checked and we summarized the data of selected manuscripts.

*Please see the "Reference List for Supplemental Tables”.

Table S3. The summery of IC50 values of Ba/F3 cells with EML4-ALK plus resistance mutations to each ALK-TKIs

[nM]

ALK Ba/F3		Crizotinib	Alectinib	Ceritinib	Brigatinib	Lorlatinib	Entrectinib	Gilteritinib	References*
EML4-ALK v1		6.05~45	0.27~23	1.7~10	0.95~10.9	1~2.3	19	0.78	S8-S12
	L1151Tins	561		91					S11
	T1151K	85~152	16	43	4.6	4.8	19	1.2	S13, S14
	L1152V	162							S13
	C1156Y	61.9~71	10~11.6	5.3~20	3.2~4.5	4.4~4.6	25	0.66	S8, S14
	I1171N	130.1~220	397.7~600	8.2~36	20~28	49~87	729	6.1	S8, S12, S14
	I1171S	65~94.1	78~177	3.8~11	7.1~17.8	30.4~38	294	2.9	S8, S14
	I1171T	33.7~413	16.6~57	1.7~14	3.82~6.8	11.5~21	196	4.2	S8, S9, S11, S13, S14
	F1174C	115~319	27	38	18	8			S8, S13
	F1174I	92	28	30	16	10	852	4.7	S14
	F1174V	68	22	20	9.3	6.2	318	3.4	S14
	V1180L	15.5~32	123~248	0.14~2.2	0.49~1.2	2.3	22	1.4	S9, S14
	L1196M	249~1215	46.4~117.6	7.5~21	14~26.5	18~73	147	20	S8, S10, S11, S13, S14
	L1196Q	181	336	42	21	33	405	26	S14
	L1198F	0.4~3.1	36~42.3	38~196.2	13.9~15	14~14.8	3.2	0.1	S8, S14
	G1202R	221~415	299.6~990	74~218	50~259.5	37.0~110	5204	168	S8, S10, S11, S12, S14
	G1202del	58.4	58.8	50.1	95.8	5.2			S8, S14
	D1203N	116.3~157	27.9~28	35.3~41	20~34.6	11.1~14	168	53	S8, S14
	S1206Y	124		1.5					S11
	S1206R	728							S13
	E1210K	42.8~297	31.6	5.8	24	1.7			S8
	F1245C	269							S13
	F1245V	38	14	13	5	2.6	127	1.4	S14
	L1256F	422	2.6	185	16	8801	2424	0.34	S14
	G1269A	98~130	18~25	0.4~3.4	1.1	10~15	59	1.4	S8, S11, S13, S14
	G1269S	953							S13
	C1156Y/L1198F	12.3	138.4	524	44.6	117			S15
	I1171N/F1174I	343~350	1188~1300	140~167	110~120	320~338	5753	24	S12, S14
	I1171N/F1174L	73~440	320~1600	32~160	18~100	37~220	1112	3.2	S12, S14
	I1171N/L1196M	299~460	229~460	12~21	23~31	355~360	379	14	S12, S14
	I1171N/L1198F	18~19	750~860	125~660	43~59	240~419	60	1.6	S12, S14
	I1171N/L1198H	100~160	799~1600	218~540	111~140	358~670	908	6.9	S12, S14
	I1171N/L1256F	398~560	37~400	73~182	18~42	5012~6000	3076	0.41	S12, S14
	I1171N/G1269A	607/670	880~1100	14	6.4~7.1	470~625	2521	11	S12, S14
	I1171S/G1269A	543	617	14	5.1	855	2446	13	S12, S14
	L1196M/L1198F	45.6	136.1	509.9	120.9	172.4			S10
	G1202R/L1196M	361~849.5	690~1148.5	318~880	190~524.9	1050~1400	1711	117	S10, S14
	G1202R/F1174C	320	700	280	180	300			S12
	G1202R/F1174L	200	560	260	160	110			S12
	G1202R/L1198F	35~74.7	296~492	299~632.1	290~1799.3	317~401	183	32	S10, S12, S14
	G1202R/G1269A	390	960	120	75	700			S12
	D1203N+F1174C	338.8	75.1	237.8	123.4	69.8			S8
	D1203N+E1210K	153	82.8	97.8	136	26.6			S8, S12
	D1203N/L1196M	304	202	140	101	266	278	109	S14
	D1203N/F1245V	170	46	87	39	30	810	64	S14
EML4-ALK v3		19~32.3	12.8	0.6~3.3	8.3	0.7			S10, S11
	L1152P	108		56					S11
	C1156Y	177		40					S11
	I1171T	106		2.6					S11
	F1174C	100		50					S11
	L1196M	200~333.1	54.4	0.9~13.3	29.9	21.2			S10, S14
	G1202R	388.6	522.9	153.5	529.5	56.9			S10
	G1269S	541		1.3					S14
	G1202R/L1196M	629	1178.5	328.8	726	1261.7			S10
	L1196M/L1198F	46.8	143.7	286.8	111.8	170.1			S10
	G1202R/L1198F	125.6	826.1	1355.7	3359.3	1160			S10

We searched in PubMed with the keywords "ALK", "lung cancer", "resistance", "Ba/F3" on March 4, 2021. Title and abstract of these manuscripts were checked and we summarized the data of selected manuscripts.

*Please see the "Reference List for Supplemental Tables”.

Table S4. The summery of IC50 values of Ba/F3 cells with ROS1 fusion plus resistance mutations to each ROS1-TKIs

[nM]

ROS1 Ba/F3		Crizotinib	Entrectinib	Loratinib	Cabozantinib	Repotrectinib	Ceritinib	Brigatinib	Taletrectinib	Alectinib	DS-6051b	References*
CD74-ROS1		2.12~44	1.7~10.5	<0.2~1	0.5~9	<0.2~2.0	10.92~70	9.4~30	2.6	302.6~>10000	0.8	S9, S16-S20
CD74-ROS1	L1951R	86.1	3	1.8	20.72		598	766.7		616.5	4.2	S9, S17
CD74-ROS1	K2003I	1.52					10.92					S9
CD74-ROS1	L2026M	56.6~259	5~3500	0.8~2	0.92		11.3~90	13.9~200		630.9~4200	3.6	S9, S16-S18
CD74-ROS1	G2032R	253.7~2700	321.1~2200	73.6~270	11.3~26	3.3~23.1	276.5~2200	170~1172	53.3	1062.2~>10000	13.5	S9, S16-S19
CD74-ROS1	D2033N	23.2~200.9	11.2~169.2	0.4~3.3	0.2	1.3	56.3~535.4	24.2~128.4		1173.3	28.3	S17, S19
CD74-ROS1	L2000V	37.1	25.9	2.5	7.6	10.1	124.9	78.9	29.8	985		S20
CD74-ROS1	L2086F	536.8	440	>3000	3.6	587.9	226.9	159.3	1265	672.5		S20
CD74-ROS1	S1986F/L2000V	159.4	36.1	2.4	5.1	7.2	86.9	62.5	20.3	1080		S20
CD74-ROS1	S1986F/L2086F	469.7	344.2	>3000	1.3	241.2	154.8	48.5	662.6	919.9		S20
CD74-ROS1	G2032R/L2086F	498.6	335.4	>3000	5	248.9	573.9	450.9	744.2	1254		S20
CD74-ROS1	S1986F/G2032R	594.4	718.5	990.6	70.1	65.1	614.7	717	105.4	1137		S20
CD74-ROS1	S1986F/G2032R/L2086F	562.8	1111	2131	9.4	1178	1116	1341	2432	1150		S20
EZR-ROS1		43		0.5			30					S21
EZR-ROS1	S1986Y	125		1			99					S21
EZR-ROS1	S1986F	116		1.6			73					S21
EZR-ROS1	G2032R	1287		508			335					S21

We searched in PubMed with the keywords "ROS1", "lung cancer" and "resistance" on March 14, 2021. Title and abstract of these manuscripts were checked and we summarized the data of selected manuscripts.

*Please see the "Reference List for Supplemental Tables”.

Reference List for Supplemental Tables

S1. Nishino M, Suda K, Kobayashi Y, et al. Effects of secondary EGFR mutations on resistance against upfront osimertinib in cells with EGFR-activating mutations in vitro. Lung Cancer. 2018; 126: 149-155.

S2. Yang Z, Yang N, Ou Q, et al. Investigating Novel Resistance Mechanisms to Third-Generation EGFR Tyrosine Kinase Inhibitor Osimertinib in Non-Small Cell Lung Cancer Patients. Clin Cancer Res. 2018; 24: 3097-3107.

S3. Uchibori K, Inase N, Nishio M, Fujita N, Katayama R. Identification of Mutation Accumulation as Resistance Mechanism Emerging in First-Line Osimertinib Treatment. J Thorac Oncol. 2018; 13: 915-925.

S4. Chiba M, Togashi Y, Bannno E, et al. Efficacy of irreversible EGFR-TKIs for the uncommon secondary resistant EGFR mutations L747S, D761Y, and T854A. BMC Cancer. 2017; 17: 281.

S5. Castellano GM, Aisner J, Burley SK, et al. A Novel Acquired Exon 20 EGFR M766Q Mutation in Lung Adenocarcinoma Mediates Osimertinib Resistance but is Sensitive to Neratinib and Poziotinib. J Thorac Oncol. 2019; 14: 1982-1988.

S6. Zhang Q, Zhang XC, Yang JJ, et al. EGFR L792H and G796R: Two Novel Mutations Mediating Resistance to the Third-Generation EGFR Tyrosine Kinase Inhibitor Osimertinib. J Thorac Oncol. 2018; 13: 1415-1421.

S7. Saxon JA, Sholl LM, Jänne PA. EGFR L858M/L861Q cis Mutations Confer Selective Sensitivity to Afatinib. J Thorac Oncol. 2017; 12: 884-889.

S8. Gainor JF, Dardaei L, Yoda S, et al. Molecular Mechanisms of Resistance to First- and Second-Generation ALK Inhibitors in ALK-Rearranged Lung Cancer. Cancer Discov. 2016; 6: 1118-1133.

S9. Katayama R, Friboulet L, Koike S, et al. Two novel ALK mutations mediate acquired resistance to the next-generation ALK inhibitor alectinib. Clin Cancer Res. 2014; 20: 5686-5696.

S10. Yoda S, Lin JJ, Lawrence MS, et al. Sequential ALK Inhibitors Can Select for Lorlatinib-Resistant Compound ALK Mutations in ALK-Positive Lung Cancer. Cancer Discov. 2018; 8: 714-729.

S11. Friboulet L, Li N, Katayama R, et al. The ALK inhibitor ceritinib overcomes crizotinib resistance in non-small cell lung cancer. Cancer Discov. 2014; 4: 662-673.

S12. Okada K, Araki M, Sakashita T, et al. Prediction of ALK mutations mediating ALK-TKIs resistance and drug re-purposing to overcome the resistance. EBioMedicine. 2019; 41: 105-119.

S13. Zhang S, Wang F, Keats J, et al. Crizotinib-resistant mutants of EML4-ALK identified through an accelerated mutagenesis screen. Chem Biol Drug Des. 2011; 78: 999-1005.

S14. Mizuta H, Okada K, Araki M, et al. Gilteritinib overcomes lorlatinib resistance in ALK-rearranged cancer. Nat Commun. 2021; 12: 1261.

S15. Shaw AT, Friboulet L, Leshchiner I, et al. Resensitization to Crizotinib by the Lorlatinib ALK Resistance Mutation L1198F. N Engl J Med. 2016; 374: 54-61.

S16. Zou HY, Li Q, Engstrom LD, et al. PF-06463922 is a potent and selective next-generation ROS1/ALK inhibitor capable of blocking crizotinib-resistant ROS1 mutations. Proc Natl Acad Sci U S A. 2015; 112: 3493-3498.

S17. Katayama R, Gong B, Togashi N, et al. The new-generation selective ROS1/NTRK inhibitor DS-6051b overcomes crizotinib resistant ROS1-G2032R mutation in preclinical models. Nat Commun. 2019; 10: 3604.

S18. Chong CR, Bahcall M, Capelletti M, et al. Identification of Existing Drugs That Effectively Target NTRK1 and ROS1 Rearrangements in Lung Cancer. Clin Cancer Res. 2017; 23: 204-213.

S19. Drilon A, Ou SI, Cho BC, et al. Repotrectinib (TPX-0005) Is a Next-Generation ROS1/TRK/ALK Inhibitor That Potently Inhibits ROS1/TRK/ALK Solvent- Front Mutations. Cancer Discov. 2018; 8: 1227-1236

S20. Lin JJ, Choudhury NJ, Yoda S, et al. Spectrum of Mechanisms of Resistance to Crizotinib and Lorlatinib in ROS1 Fusion-Positive Lung Cancer. Clin Cancer Res. 2021; 27: 2899-2909.

S21. Facchinetti F, Loriot Y, Kuo MS, et al. Crizotinib-Resistant ROS1 Mutations Reveal a Predictive Kinase Inhibitor Sensitivity Model for ROS1- and ALK-Rearranged Lung Cancers. Clin Cancer Res. 2016; 22: 5983-5991.
